# Diversity of Anal HPV and Non-HPV Sexually Transmitted Infections and Concordance with Genital Infections in HIV-Infected and HIV-Uninfected Women in the Tapajós Region, Amazon, Brazil

**DOI:** 10.3390/v15061328

**Published:** 2023-06-06

**Authors:** Luana Lorena Silva Rodrigues, José Henrique Pilotto, Katrini Guidolini Martinelli, Alcina F. Nicol, Vanessa Salete De Paula, Tarik Gheit, Nathália Silva Carlos Oliveira, Carlos Silva-de-Jesus, Vikrant V. Sahasrabuddhe, Diane M. Da Silva, W. Martin Kast, Justin Hardick, Charlotte A. Gaydos, Mariza Gonçalves Morgado

**Affiliations:** 1 Programa de Pós-Graduação em Ciências da Saúde, Instituto de Saúde Coletiva, Universidade Federal do Oeste do Pará, Santarém 68135-110, Brazil; 2 Laboratório de AIDS e Imunologia Molecular, IOC-FIOCRUZ, Rio de Janeiro 21040-360, Brazil; pilotto@ioc.fiocruz.br (J.H.P.); carlossilje@ioc.fiocruz.br (C.S.-d.-J.); 3 Programa de Pós-Graduação em Medicina Tropical, IOC-FIOCRUZ, Rio de Janeiro 21040-360, Brazil; 4 Departamento de Medicina Social, Universidade Federal do Espírito Santo, Vitória 29075-910, Brazil; katrigm@gmail.com; 5 Instituto Nacional de Infectologia Evandro Chagas, INI-FIOCRUZ, Rio de Janeiro 21040-360, Brazil; afnnicol@gmail.com; 6 Laboratório de Virologia Molecular e Parasitologia, IOC-FIOCRUZ, Rio de Janeiro 21040-360, Brazil; vdepaula@ioc.fiocruz.br; 7 International Agency for Research on Cancer, 69366 Lyon, France; gheitt@iarc.who.int; 8 Departamento de Patologia, Faculdade de Medicina, Universidade Federal Fluminense, Rio de Janeiro 23033-900, Brazil; nsoliveira95@gmail.com; 9 Division of Cancer Prevention, National Cancer Institute, Bethesda, MD 20892, USA; vikrant.sahasrabuddhe@nih.gov; 10 Department of Obstetrics and Gynecology, University of Southern California, Los Angeles, CA 90033, USA; diane.dasilva@med.usc.edu (D.M.D.S.); martin.kast@med.usc.edu (W.M.K.); 11 Norris Comprehensive Cancer Center, University of Southern California, Los Angeles, CA 90089, USA; 12 Department of Molecular Microbiology and Immunology, University of Southern California, Los Angeles, CA 90089, USA; 13 Division of Infectious Diseases, Johns Hopkins University School of Medicine, Baltimore, MD 21205, USA; jhardic1@jhmi.edu (J.H.); cgaydos@jhmi.edu (C.A.G.)

**Keywords:** HPV, STI, anal and cervical infectious, immune responses

## Abstract

The aim of this study was to classify the diversity of anal HPV and non-HPV sexually transmitted infections (STIs) and compare the concordance between anal and genital infections in HIV-infected and uninfected women living in the Tapajós region, Amazon, Brazil. A cross-sectional study was performed with 112 HIV-uninfected and 41 HIV-infected nonindigenous women. Anal and cervical scrapings were collected and analyzed for HPV, *Chlamydia trachomatis* (CT)*, Neisseria gonorrheae* (NG), *Trichomonas vaginalis* (TV), *Mycoplasma genitalium* (MG), and *Human alphaherpesvirus* 2 (HSV-2). The Kappa test evaluated the concordance between anal and genital infections. The overall prevalence of anal HPV infection was 31.3% in HIV-uninfected and 97.6% in HIV-infected women. The most frequent anal high-risk HPV (hrHPV) types were HPV18 and HPV16 in HIV-uninfected women and HPV51, HPV59, HPV31, and HPV58 in HIV-infected women. Anal HPV75 *Betapapillomavirus* was also identified. Anal non-HPV STIs were identified in 13.0% of all participants. The concordance analysis was fair for CT, MG, and HSV-2, almost perfect agreement for NG, moderate for HPV, and variable for the most frequent anal hrHPV types. Thus, a high prevalence of anal HPV infection with moderate and fair concordance between anal and genital HPV and non-HPV STIs was observed in our study.

## 1. Introduction

Data on sexually transmitted infections (STIs) are predominantly related to genital infections. Studies on the anal human papillomavirus (HPV) and non-HPV STIs remain scarce. Cervical cancer screening might contribute to the prevention of anal cancer in women as a secondary prevention measure for anal cancer when stratified to HIV-uninfected women older than 45 years, those with high-risk HPV (hrHPV) cervical infection, or HIV-infected women [1]. Changes in sexual practices and risky behavior trends can increase the prevalence of anal STIs in the developed world [2]. Transmission between the anus and cervix and vice versa are common in women [3,4,5]. This aspect needs to be further investigated because the woman may have HPV infection in both sites, genital and anal, at the same time that it is investigated, but which may be of different HPV types acquired in different exposures in life. The genital site is likely the main source of a concurrent genital–anal HPV infection, which is well mentioned in the scientific literature [4], but it is a hypothesis supported by studies with limitations [6]. Concordance studies between genital and anal infections and the HPV genotypes identified between the two sites can bring clearer answers if they use more robust methodological approaches for HPV detection and genotyping, as well as statistics with better robustness [6]. People living with HIV, men who have sex with men (MSM), and women with cervical cancer are at risk for anal cancer [3]. Recently, age-specific shifts in HPV16 prevalence from the cervix to the anus have been demonstrated, suggesting that HPV infections in the anus persist longer or occur later in life than those in the cervix, particularly in HIV-infected women [7]. Women living in the Tapajós region of the Amazon have a high prevalence of cervical HPV infection and diverse HPV types, along with other STIs [8,9,10]. Therefore, the present study aimed to identify the prevalence and diversity of anal HPV and non-HPV infections and assess the concordance between anal and genital HPV and non-HPV STIs in HIV-uninfected and HIV-infected women. The results obtained in the present study are of relevance when evaluating the utility of cervical screening results in stratifying anal cancer risk.

## 2. Materials and Methods

### 2.1. Ethical Issues

Women were invited to participate in the study after signing a written informed consent document in strict compliance with the Brazilian ethical guidelines involving research on human subjects (protocol numbers 2.013.163 and 2.121.697). An epidemiological interview was then conducted.

### 2.2. Study Design and Population

This study was supported by previous studies in which we evaluated the acceptability of cervico-vaginal self-collection, HPV and other STI testing, and sociodemographic data of 153 nonindigenous women, 112 HIV-uninfected and 41 HIV-infected, living in the Tapajós region, fully described elsewhere [8,9,10]. For the present study, we added the molecular characterization of anal HPV and non-HPV infections in the same study group.

### 2.3. Anal and Cervical Samples

Anal scraping was performed using a brush introduced approximately 1.95 inches into the anal canal, rotated 680° five times, and later preserved in *ThinPrep* Solution. Anal and cervical sample collection and processing, as well as the transportation of the biological samples according to international rules (IATA) for laboratory analyses, are described elsewhere [5,6,7].

### 2.4. DNA Extraction and HPV DNA Detection

DNA extraction from the anal samples was performed with the QIAamp DNA Mini Kit (Qiagen, Valencia, CA, USA) according to the manufacturer’s protocol and was quantified by spectrophotometry on a NanoDrop (ND 1000, Fisher Scientific, Wilmington, DC, USA). Nested PCR was performed to increase the specificity of HPV DNA detection, with PGMY09 and PGMY11 primers for the first round and GP5+ and GP6+ primers for the second round, as previously described [8], in an automatic thermocycler (GeneAmp PCR System 9700—Applied Biosystems).

### 2.5. HPV Genotyping and Multiple HPV Types

Nested PCR products were purified with a Wizard^®^ SV Gel and PCR Clean-up System (Promega Madison, WI, USA). HPV genotyping and multiple HPV types were performed according to previously published protocols [8,11]. HPV samples were grouped into three groups considering the oncogenic risk classification as follows: high-risk, “probable” or “possible” carcinogenic, and low-risk [12]. The only positive anal scraping sample for *Betapapillomavirus* was identified through nucleotide sequencing analysis and confirmed at the International Agency for Research on Cancer in Lyon, France, by type-specific multiplex genotyping (TS-MPG) assays combining multiplex polymerase chain reaction (PCR) and bead-based Luminex technology (Luminex Corp., Austin, TX, USA), as described elsewhere [13,14].

### 2.6. Non-HPV STI Molecular Detection

In this study, the non-HPV STIs assessed were *Chlamydia trachomatis* (CT), *Neisseria gonorrheae* (NG), *Trichomonas vaginalis* (TV), *Mycoplasma genitalium* (MG), and *Human alphaherpesvirus* 2 (HSV-2 or herpes simplex virus 2). Duplex real-time PCR was performed for CT and NG, and three real-time PCRs were performed independently to detect TV, MG, and HSV-2. The molecular detection of other non-HPV STIs was performed as previously described [9,10]. All reactions were performed in a QuantStudio 12 K Flex Real-Time PCR System (Applied BioSystems by Life Technologies, Carlsbad, CA, USA).

### 2.7. Statistical Analysis

Descriptive statistics of the qualitative variables were determined by frequency distribution. The Chi-square test at 95% CI and *p*-value ≤ 0.05 was used to compare the proportions between the groups. A Mann–Whitney test with 95% CI and *p*-value ≤ 0.05 was performed to evaluate the associations among median HIV viral load, CD4+ T-cell count, and anal hrHPV infection. Pearson’s correlation test with 95% CI and *p*-value ≤ 0.05 was performed to evaluate the association of the quantitative proportion of the CD4+ T-cell count and the number of distinct hrHPV types. A Kappa test with 95% CI and *p*-value ≤ 0.05 was performed to evaluate the concordance between anal and genital HPV and other STIs (non-HPV STIs; CT, NG, TV, MG, and HSV-2) [15] isolated from anal and cervical scraping, respectively [8,9,10]. We categorized the absence and presence of high- and low-risk HPV types, the absence or presence of non-HPV STIs, and the absence and presence of the most frequent anal hrHPV types to perform the proposed Kappa test. Probable and possible carcinogenic HPV types were considered as low-risk HPV for the Kappa test. Statistical analyses were performed using the Statistical Package for Social Sciences (SPSS) software version 19.0 (IBM Corp., Armonk, NY, USA).

## 3. Results

### 3.1. Main Sociodemographic Characteristics of the Participants

A total of 153 (N) women agreed to participate in the study, and their anal and cervical scrapings (clinician-collected samples) were collected on the same day. The participants were divided into two groups: HIV-uninfected women (*n* = 112) and HIV-infected women (*n* = 41). The mean age of all participants was 36.9 (±12.9) years. Most of the HIV-infected women were single (*p* = 0.006), had an average of 4.3 gestations (*p* = 0.050), started sex at the age of 17 years or below (*p* = 0.052), had more than four sexual partners in life (*p* = 0.012), and regularly used condoms (*p* = 0.010) (Table 1). The practice of anal sex (*p* = 0.457) did not significantly differ between the two groups (Table 1).

### 3.2. Anal HPV Infection and Diversity

**Figure 1:**
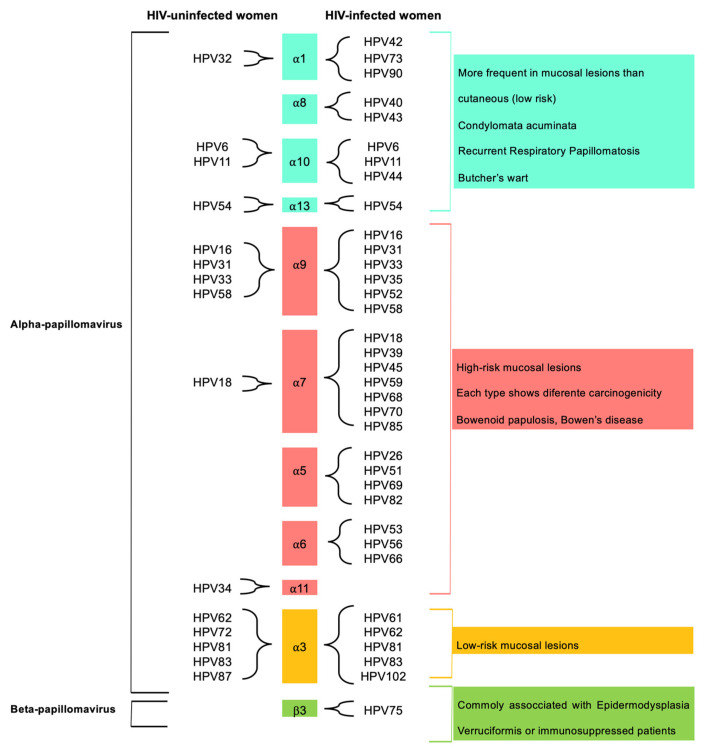
Representative diversity of anal HPV genotypes by genus, with *Alphapapillomavirus* and *Betapapillomavirus* identified in HIV-uninfected and HIV-infected women, and the predominant tissue associations and typical relationships between HPV type and disease caused as proposed in [12,16].

The overall prevalence of anal HPV infection was 31.3% (35/112) among HIV-uninfected women and 97.6% (40/41) among HIV-infected women. A high diversity of anal HPV genotypes was identified in HIV-uninfected and HIV-infected women participating in the study (Figure 1). Anal HPV types of the genus *Alphapapillomavirus* (97.4%; 38/39) and *Betapapillomavirus* (2.6%; 1/39) are presented in Figure 1, where we also included the predominant tissue associations and the typical relationships between HPV type and disease, as proposed in [12,16].

### 3.3. Anal hrHPV and Sociodemographic Characteristics of HIV-Uninfected and HIV-Infected Women in the Tapajós Region, Amazon, Brazil

The groups of HIV-uninfected women (*n* = 112) and HIV-infected women (*n* = 41) were further analyzed according to the presence or absence of anal hrHPV, based on sociodemographic characteristics. Among HIV-uninfected women, most of those with anal hrHPV had more than four lifetime sexual partners (*p* = 0.040) and cervical HPV (*p* = 0.045), whereas among HIV-infected women, only cervical hrHPV was significantly associated with anal hrHPV (*p* ≤ 0.001). The practice of anal sex was not significant for the occurrence of anal hrHPV in both groups of women infected and not with HIV (Table 2).

### 3.4. Single and Multiple Anal HPV Infections and HPV Types in HIV-Uninfected and HIV-Infected Women

Among the HIV-uninfected women, 97.0% (34/35) had single anal HPV infections, and 3.0% (1/35) had multiple anal HPV infections. In HIV-infected women, multiple anal HPV infections were much more prevalent, at 85.0% (34/40), and single anal HPV infections accounted for 15.0% (6/40).

**Figure 2:**
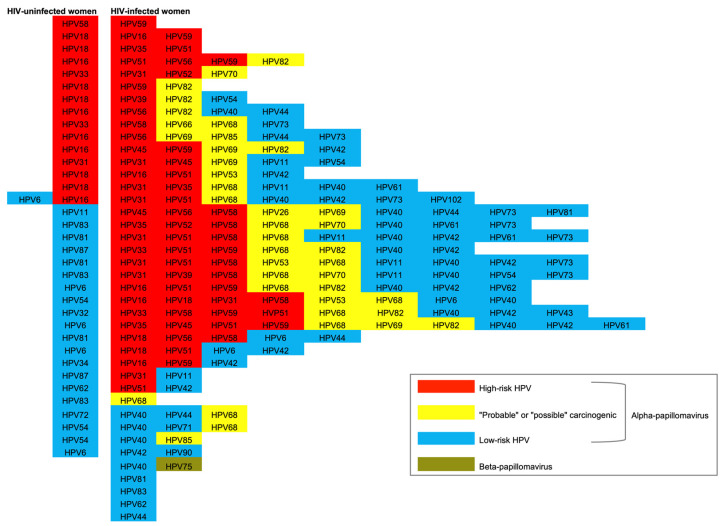
Diversity of anal HPV types isolated from HIV-uninfected and HIV-infected women. The columns are not organized by paired samples from the same women but according to the oncogenic risk classification of *Alphapapillomavirus*. The high-risk HPV types are shown in red, “probable” or “possible” carcinogenic in yellow, and low-risk in blue. *Betapapillomavirus* is shown in green.

Anal HPV types found in the participants were predominantly of the genus *Alphapapillomavirus*. Based on the oncogenic risk classification, the HPV samples were grouped into high-risk, “probable” or “possible” carcinogenic, and low-risk groups (Figure 2). Anal hrHPV infections were significantly more prevalent in HIV-infected women than in HIV-uninfected women (Chi-square test *p* < 0.001).

The overall prevalence of hrHPV was 42.9% (15/35) among HIV-uninfected women and 75.0% (30/40) among HIV-infected women. HPV18 (*n* = 6) and HPV16 (*n* = 5) were the most frequent anal hrHPV types in HIV-uninfected women, representing 73.3% (11/15) of the total anal hrHPV types genotyped in this group. The most frequent anal hrHPV types in HIV-infected women were HPV51 (*n* = 12), HPV59 (*n* = 10), HPV31 (*n* = 9), and HPV58 (*n* = 9).

All HIV-infected women were on combination antiretroviral therapy (cART). There was no significant association between anal hrHPV infection, median HIV viral load (Mann–Whitney test *p* = 0.110), and median CD4+ counts (Mann–Whitney test *p* = 0.828). Pearson’s correlation test shows that HIV-infected women with lower CD4+ counts do not carry a significantly higher number of distinct anal hrHPV types (rô = −0.179; *p* = 0.262).

### 3.5. Anal Non-HPV STIs in HIV-Uninfected and HIV-Infected Women

**Figure 3:**
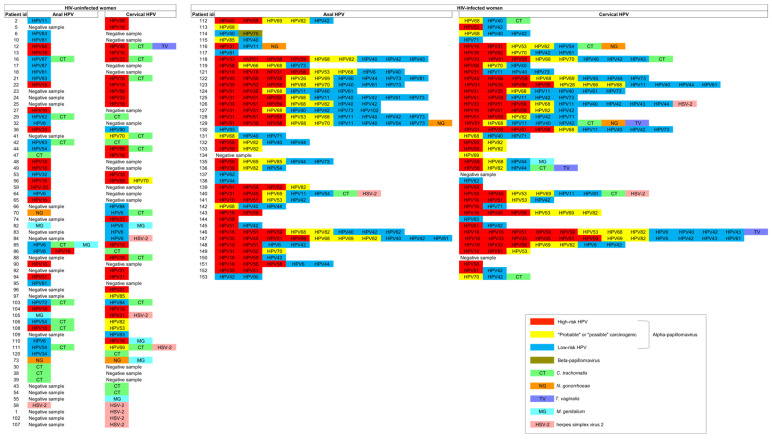
Comparison between the anal and genital HPV types and non-HPV STIs isolated from anal and cervical scrapings collected simultaneously from the same patient. The columns are organized by paired samples from the same women.

Anal non-HPV STIs were identified in 15.2% (17/112) of HIV-uninfected women and 7.3% (3/41) of HIV-infected women (Figure 3). The prevalence of CT, NG, MG, TV, and HSV-2 in the anal compartment of HIV-uninfected and HIV-infected women has already been published elsewhere [7].

### 3.6. Concordance between Anal and Genital HPV and Non-HPV STIs in HIV-Uninfected and HIV-Infected Women

Genital HPV infection and other STIs observed in the groups of women included in the present study have already been published by our group [8,9,10]. The comparison between the anal and genital microbiota of all women revealed differences between the HPV types and non-HPV STIs isolated from the anal and cervical scrapings collected simultaneously from the same patient (Figure 3). The Kappa test was performed for concordance analysis between anal and genital HPV infection and non-HPV STIs in HIV-uninfected and HIV-infected women. We categorized the absence and presence of high- and low-risk HPV types, the absence or presence of non-HPV STIs, and the absence and presence of the most frequent anal hrHPV types to perform the proposed Kappa test (Table 3). The concordance analysis was fair for CT, MG, and HSV-2, with Kappa values of 0.26, 0.23, and 0.35, respectively; moderate for HPV, considering the oncogenic risk classification, with a Kappa value of 0.44; and in almost perfect agreement for NG, with a Kappa value of 0.85 (Table 3). The Kappa test could not be conducted for TV due to a lack of agreement in the positive results across samples from the same women. Considering the most frequent anal hrHPV types for all women participants, the concordance analysis was poor for HPV16 (Kappa value 0.18), fair for HPV18 (Kappa value 0.34) and HPV31 (Kappa value 0.38), moderate for HPV58 (Kappa value 0.57), and HPV59 (Kappa value 0.41) and substantial for HPV51 (Kappa value 0.78) (Table 3). By group, for HIV-uninfected women, the concordance analysis was no agrément for HPV16 (Kappa value −0.05) and fair for HPV18 (Kappa value 0.37), and for HIV-infected women, fair for HPV31 (Kappa value 0.32) and HPV59 (Kappa value 0.29) and substantial for HPV51 (Kappa value 0.71) and HPV58 (Kappa value 0.69) (Table 3).

## 4. Discussion

In the present study, a high overall prevalence of anal HPV infection was identified in both HIV-uninfected (31.3%) and HIV-infected (97.6%) women living in the Tapajós region, higher than the national prevalence of anal HPV infection (25.68%) [17]. Moreover, greater diversity and multiplicity of anal HPV genotypes, including the *Betapillomavirus* genus, were detected (Figure 1).

Our earlier analyses revealed that this cohort of women had a high prevalence of cervical HPV infection, hrHPV, and other STIs [8,9,10]. However, the presence of cervical dysplasias (cervical intraepithelial lesions) did not differ significantly among them [8] and is close to that found in women with abnormal cytology (54.2%) referred to a colposcopy/cervical pathology clinic in Greece [18].

In this study, a high overall prevalence of anal HPV infection was identified in HIV-uninfected and HIV-infected women. However, the prevalence of anal hrHPV infection, despite the practice of anal sex, did not differ significantly among the participants (Table 1). Furthermore, having cervical hrHPV was significantly associated with the presence of anal hrHPV in both HIV-uninfected and HIV-infected groups (Table 2). These findings corroborate the hypothesis that the transmission of HPV between the anus and cervix and vice versa is common in women [3], and there is a high risk of acquiring an anal HPV infection after cervical infection with HPV of the same genotype, which can occur independently of anal sex intercourse [19,20]. Cervical hrHPV infection is a risk factor for acquiring anal hrHPV infection in women with or without cervical intraepithelial lesions, although the prevalence of anal and cervical hrHPV infection identified in this study was very similar, being 42.9% and 47.9% for HIV-uninfected women and 75.0% and 77.5% for HIV-infected women, respectively [21,22].

No significant association was found between anal hrHPV infection and median HIV viral load or median CD4+ count. Although lower CD4+ counts did not lead to a significantly higher number of distinct anal hrHPV types, anal hrHPV infections were significantly more prevalent in HIV-infected women than in HIV-uninfected women (Chi-square test p < 0.001). Persistent hrHPV infection is directly associated with cancer development due to the accumulation of alterations in numerous cellular processes, including DNA repair, angiogenesis, or apoptosis caused by the viral oncogenes E6 and E7 [23,24]. In addition, immunosuppression associated with HIV infection and other STIs makes it difficult to control oncogenic viral processes, increasing the risk of hrHPV-associated anal cancer [25]. High diversity and multiplicity of HPV types in HIV-uninfected and HIV-infected women were identified in anal samples, moderate concordance between anal and genital HPV in the two groups of women was identified (Kappa value 0.44) and variable concordance between anal and genital HPV in the most frequent anal hrHPV types among all participants and by group of women (Table 3). The high diversity and multiplicity of HPV types in anal samples and moderate concordance between anal and genital HPV observed in this study are important because these women were screened simultaneously with a molecular test to detect HPV DNA by conventional PCR, multiplex assays and nucleotide sequencing of the anal and genital samples, which suggests that at different times in life, these women may have contracted different HPV genotypes. Thus, the results do not simply reflect the concomitant or consecutive transmission between the anus and cervix. A fair concordance between anal and genital non-HPV STIs in HIV-uninfected and HIV-infected women was also identified. This result aligns with previous findings because CT, MG, and HSV-2 (Kappa values of 0.26, 0.23, and 0.35, respectively) have higher tropism for cervical epithelial cells, considering the natural history of the disease [26,27].

Our study has some limitations. The cross-sectional epidemiological design does not allow the temporal relationship between exposure and effect to be established, making it difficult to establish a causal relationship. The impediment to performing the Kappa test for concordance analysis for TV and the difficulty in interpreting the results of non-HPV STIs may have occurred due to the low prevalence of other STIs, potentially influenced by the sample size. In addition, as a limitation for performing the Kappa test for concordance analyses, we categorized HPV types as high- and low-risk and the most frequent anal hrHPV types, rather than HPV genotypes, due to the great variability of HPV types observed in both groups, which would reduce the statistical power of the comparative analyses. This is a limitation of this type of comparative study when there is a great diversity of identified HPV genotypes.

This high prevalence of anal HPV and hrHPV infections and the high diversity and multiplicity of HPV types identified in HIV-uninfected and HIV-infected women in the Tapajós region add to the growing body of evidence published in the scientific literature that the high prevalence and long-term persistence of HPV infection is a hereditary characteristic of the prehistoric people of the Amazon [8,28,29]. Those living farther away from the Amazon urban centers are more likely to be infected by hrHPV.

Recent studies have suggested that hrHPV infection is a sufficient cause of anal intraepithelial lesions and anal cancer [23,24,30]. However, others have suggested that HPV high intrahost mutation rates are associated with the anatomical site, HIV coinfection, and the possibility of syndemic interactions [25,30,31]. Further studies on the anogenital microbiome could expand our knowledge about HPV, carcinogenesis, and the role of cofactors in this process, such as non-HPV STIs, including HIV.

The early onset of sexual activity (sex at the age of 17 years or under), a high number of pregnancies (3.4 gestations on average), the number of sexual partners (up to four sexual partners in life), and the nonuse of condoms are possibly the main risk factors related to the high prevalence and diversity of anal HPV types identified in the two groups of women included in the present study. Social factors, such as difficult access to communities and lack of access to basic public health services, have a higher impact on the consequences of long-term persistent anal HPV infection for women. Cultural and behavioral factors, such as low frequency of condom usage and limited hygienic conditions, might explain recurrent infections associated with high HPV prevalence and diversity [32]. In addition, the high prevalence of anal HPV and hrHPV found in this study may be related to the high sensitivity and specificity of the laboratory techniques used for HPV genotyping, as already mentioned in [8].

In agreement with the literature [1,7,33], HPV16 and HPV18 were the most prevalent anal hrHPV types found in HIV-uninfected women. The Ts group of women also had a high prevalence of HPV16 and HPV18 in their cervicovaginal specimens [8]. It may be essential to pay attention to the hypothesis raised by systematic reviews and meta-analyses that HIV-uninfected women with cervical HPV16, especially those older than 45 years, have a similar anal cancer risk profile to that of HIV-infected women [1,7,33]. Therefore, these women are high-risk target groups for secondary anal cancer prevention.

The most frequent anal hrHPV types identified in HIV-infected women included in our study were HPV51, HPV59, HPV31, and HPV58, which are HPV types not covered by the 4-valent or 2-valent HPV vaccines available in Brazil. HPV16, followed by HPV18, are the types most related to anal cancer; however, HPV31, HPV33, HPV45, HPV52, and HPV58 were extracted from the nonHPV16 fraction and are possibly associated with anal cancer [27]. This study reinforces the detection of HPV31 and HPV58 as described in other studies in the anus [27] and described in the literature as a nonHPV16 fraction possibly attributed to anal cancer and further verifies the relationship between HPV51 and HPV59 types that can be attributed to anal cancer. Therefore, these HPV types should be considered for incorporation into a broad-spectrum vaccine for HPV types of clinical importance. To our knowledge, this is the first study on anal HPV infection that compared the concordance between anal and genital infection with HPV and non-HPV STIs on the prevalence and genotyping of anal HPV and the concordance with genital infection in the general population of women in northern Brazil [17]. Compared to HIV-infected women living in other populations [31,34], those living in the Tapajós region have different types of circulating anal hrHPV types with greater diversity and multiplicity of anal HPV types, despite taking cART, being clinically and immunologically well and exhibiting virological suppression. Understanding whether the different types of HPV are acquired independently of one another has implications for the natural history of HPV-related disease and the effectiveness of the prophylactic HPV vaccine. Considering the antagonism or synergism between the viral types in the infection of target tissues would improve the replacement of vaccine types with competing species [20].

The Tapajós region, Amazon, located in northern Brazil, has the highest cervical cancer incidence and mortality rates among women in a national ranking. It is necessary to consider reviewing and updating public health policies on cervical cancer screening and furthering the fight against HIV/AIDS and other STIs. New technologies are warranted for screening and achieving early diagnoses, such as alternative self-collection samples for HPV and non-HPV STIs and DNA testing based on nucleic acid amplification assays (NAATs). Altogether, these approaches might enable a better assessment of cervical screening results to stratify anal cancer risk in high-risk groups, irrespective of HIV status, and to expand cancer screening services to populations in limited-resource settings and remote geographical regions.

## 5. Conclusions

Overall, the prevalence of anal HPV infection is high in HIV-uninfected and HIV-infected women living in the Tapajós region of the Amazon. We identified a much higher prevalence rate of anal HPV and hrHPV than those reported in the literature for the Brazilian population, with different types of hrHPV not included in the 4-valent HPV vaccine. There was a greater diversity and multiplicity of anal HPV types, particularly in HIV-infected women. Moderate and fair concordance between anal and genital HPV infection and non-HPV STIs were identified, respectively. We encourage public health care officials to consider stratifying anal cancer risk in high-risk groups, irrespective of HIV status, when assessing cervical screening results, particularly in regions where there is a high prevalence of anal HPV and hrHPV infection with high diversity and multiplicity of types.

## Figures and Tables

**Table 1:** Main sociodemographic characteristics of HIV-uninfected and HIV-infected women in the Tapajós region, Amazon, Brazil.

Variables	HIV-Uninfected *n* = 112 (%)	HIV-Infected *n* = 41 (%)	Total N = 153 (%)	*p*-Value *
Age range (years)				0.242
≤20	08 (7.1)	05 (12.2)	13 (8.5)	
21–30	36 (32.1)	08 (19.5)	44 (28.8)	
≥31	68 (60.7)	28 (68.3)	96 (62.7)	
Marital status				0.006
Single	36 (32.7)	23 (57.5)	59 (39.3)	
Married/living together	74 (67.3)	17 (42.5)	91 (60.7)	
Number of pregnancies	3.0 (±2.3)	4.3 (± 5.7)	3.4 (± 3.5)	0.050 #
Age at first sexual intercourse (years)				0.052
≤17	71 (64.0)	33 (80.5)	104 (68.4)	
≥18	40 (36.0)	08 (19.5)	48 (31.6)	
Number of sexual partners				0.012
1–3	57 (51.4)	11 (28.2)	68 (45.3)	
≥4	54 (48.6)	28 (71.8)	82 (54.7)	
Regular use of condoms				0.010
Yes	13 (11.7)	12 (29.3)	25 (16.4)	
No	98 (88.3)	29 (70.7)	127 (83.6)	
Anal sex practice				0.457
Yes	67 (60.4)	22 (53.7)	89 (58.6)	
No	44 (39.6)	19 (46.3)	63 (41.4)	

* Chi-square test result. # *T*-student test result.

**Table 2:** Anal high-risk HPV according to sociodemographic characteristics of HIV-uninfected and HIV-infected women in the Tapajós region, Amazon, Brazil.

Variables	Anal hrHPV			
	Yes N (%)	No N (%)	Total (%)	*p*-Value *
**HIV-uninfected (*n* = 112)**				
Age range (years)				0.510
≤20	0 (0.0)	08 (8.2)	08 (7.1)	
21–30	05 (33.3)	31 (32.0)	36 (32.1)	
≥31	10 (66.7)	58 (59.8)	68 (60.7)	
Marital status				0.560
Single	06 (40.0)	30 (31.6)	36 (32.7)	
Married/ living together	09 (60.0)	65 (68.4)	74 (67.3)	
Age of first sexual intercourse (years)				0.164
≤17	12 (80.0)	59 (61.5)	71 (64.0)	
≥18	03 (20.0)	37 (38.5)	40 (36.0)	
Number of sexual partners				0.040
1–3	04 (26.7)	53 (55.2)	57 (51.4)	
≥4	11 (73.3)	43 (44.8)	54 (48.6)	
Regular use of condoms				0.050
Yes	04 (26.7)	09 (9.4)	13 (11.7)	
No	11 (73.3)	87 (90.6)	98 (88.3)	
Anal sex practice				0.591
Yes	10 (66.7)	57 (59.4)	67 (60.4)	
No	05 (33.3)	39 (40.6)	44 (39.6)	
Cervical hrHPV				0.045
Yes	06 (40.0)	17 (17.5)	23 (20.5)	
No	09 (60.0)	80 (82.5)	89 (79.5)	
**HIV-infected (*n* = 41)**				
Age range (years)				0.916
≤20	04 (13.3)	01 (9.1)	05 (12.2)	
21–30	06 (20.0)	02 (18.2)	08 (19.5)	
≥31	20 (66.7)	08 (72.7)	28 (68.3)	
Marital status				0.730
Single	16 (55.2)	07 (63.6)	23 (57.5)	
Married/ living together	13 (44.8)	04 (36.4)	17 (42.5)	
Age of first sexual intercourse (years)				0.412
≤17	23 (76.7)	10 (90.9)	33 (80.5)	
≥18	07 (23.3)	01 (9.1)	08 (19.5)	
Number of sexual partners				0.935
1–3	08 (28.6)	03 (27.3)	68 (45.3)	
≥4	20 (71.4)	08 (72.7)	82 (54.7)	
Regular use of condoms				0.701
Yes	08 (26.7)	04 (36.4)	12 (29.3)	
No	22 (73.3)	07 (63.6)	29 (70.7)	
Anal sex practice				0.524
Yes	17 (56.7)	05 (45.5)	22 (53.7)	
No	13 (43.3)	06 (54.5)	19 (46.3)	
Cervical hrHPV				0.001
Yes	27 (90.0)	04 (36.4)	31 (75.6)	
No	03 (10.0)	07 (63.6)	10 (24.4)	

* Chi-square test result.

**Table 3:** Concordance of anal and genital HPV, non-HPV STIs, and the most frequent anal high-risk HPV types isolated from HIV-uninfected and HIV-infected women using the Kappa test.

STIs	HPV	CT	NG	MG	HSV-2	TV
All participants (N = 153)						
Agreement	66.01%	84.96%	99.34%	96.07%	95.42%	- *
Positive	101/153	130/153	152/153	147/153	146/153	- *
Kappa (95% CI)	0.44 (0.33–0.56)	0.26 (0.05–0.47)	0.85 (0.45–1.00)	0.23 (−0.03–0.66)	0.35 (0.00–0.72)	- *
*p*-value	<0.001	0.001	<0.001	0.003	<0.001	- *
HIV-uninfected (*n* = 112)						- *
Agreement	61.61%	84.82%	99.10%	95.53%	94.64%	- *
Positive	69/112	95/112	111/112	107/112	106/112	- *
Kappa (95% CI)	0.22 (0.07–0.37)	0.29 (0.02–0.51)	0.66 (0.04–1.00)	0.26 (−0.04–0.66)	0.24 (0.00–0.66)	- *
*p*-value	0.002	0.002	<0.001	0.005	<0.001	- *
HIV-infected (*n* = 41)						- *
Agreement	78.05%	85.36%	100%	- *	97.56%	- *
Positive	32/41	35/41	41/41	- *	40/41	- *
Kappa (95% CI)	0.45 (0.16–0.72)	0.22 (0.0–0.64)	1.00	- *	0.65 (0.00–1.00)	- *
*p*-value	0.001	0.026	<0.001	- *	<0.001	- *
**The most frequent anal hrHPV types**	**HPV16**	**HPV18**	**HPV31**	**HPV51**	**HPV58**	**HPV59**
All participants (N = 153)						
Agreement	88.23%	93.46%	92.81%	96.73%	94.77%	93.46%
Positive	135/153	143/153	142/153	148/153	145/153	143/153
Kappa (95% CI)	0.18 (−0.57–0.44)	0.34 (−0.03–0.61)	0.38 (0.06–0.65)	0.78 (0.56–0.94)	0.57 (0.25–0.81)	0.41 (0.07–0.68)
*p*-value	0.018	<0.001	<0.001	<0.001	<0.001	<0.001
HIV-uninfected (*n* = 112)						
Agreement	89.28%	94,64%				
Positive	100/112	106/112				
Kappa (95% CI)	−0.05 (−0.08–0.16)	0.37 (−0.03–0.78)				
*p*-value	0.555	<0.001				
HIV-infected (*n* = 41)						
Agreement			80.48%	87.80%	90.24%	75.60%
Positive			33/41	36/41	37/41	31/41
Kappa (95% CI)			0.32 (−0.84–0.66)	0.71 (0.43–0.93)	0.69 (0.32–0.93)	0.29 (−0.09–0.62)
*p*-value			0.028	<0.001	<0.001	0.06

* It was not possible to perform the Kappa test because there was no agreement between the positive test and the molecular detection results from samples from the same woman. CT = *Chlamydia trachomatis*, NG = *Neisseria gonorrheae*, MG = *Mycoplasma genitalium*, HSV-2 = herpes simplex virus 2, and TV = *Trichomonas vaginalis*.

## Data Availability

The data are contained within the article.
